# Supplementary material for: Cytosine modifications exhibit circadian oscillations that are involved in epigenetic diversity and aging
Source: Nat Commun. 2018 Feb 13;9:644. doi: 10.1038/s41467-018-03073-7 (PMC5811577; doi:10.1038/s41467-018-03073-7)
Supplement: Supplementary file 1 — Supplementary Information [file 41467_2018_3073_MOESM1_ESM.pdf]

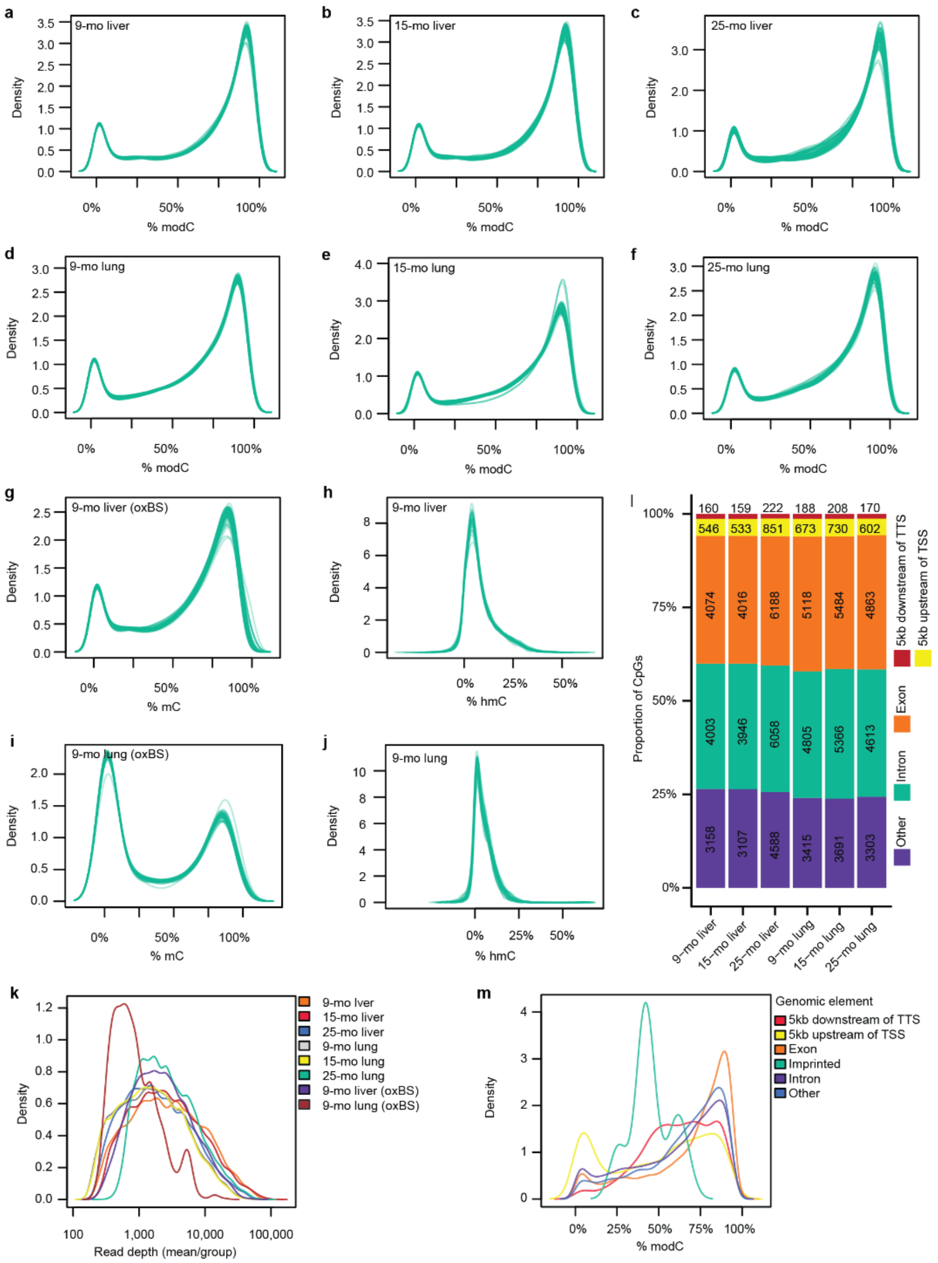

Supplementary Figure 1. Quality control for bisulfite and oxidative bisulfite padlock sequencing of chromosome 7 in the mouse liver and lung tissues. The cytosine modification density plots for 9-mo, 15-mo, and 25-mo mice for (a-c) the liver and (d-f) the lung. g-j) mC and hmC density plots from (g-h) the 9-mo mouse liver and (j-i) the lung. k) Density plot of the read depth of interrogated cytosines in each age and tissue group. l) Distribution of the epigenetically variable cytosines across genomic elements. m) Density of the mean cytosine modification across all bisulfite padlock datasets stratified by genomic elements. modC, modified cytosines; mC, 5-methylcytosine; hmC, 5-hydroxymethylcytosine; mo, month-old.

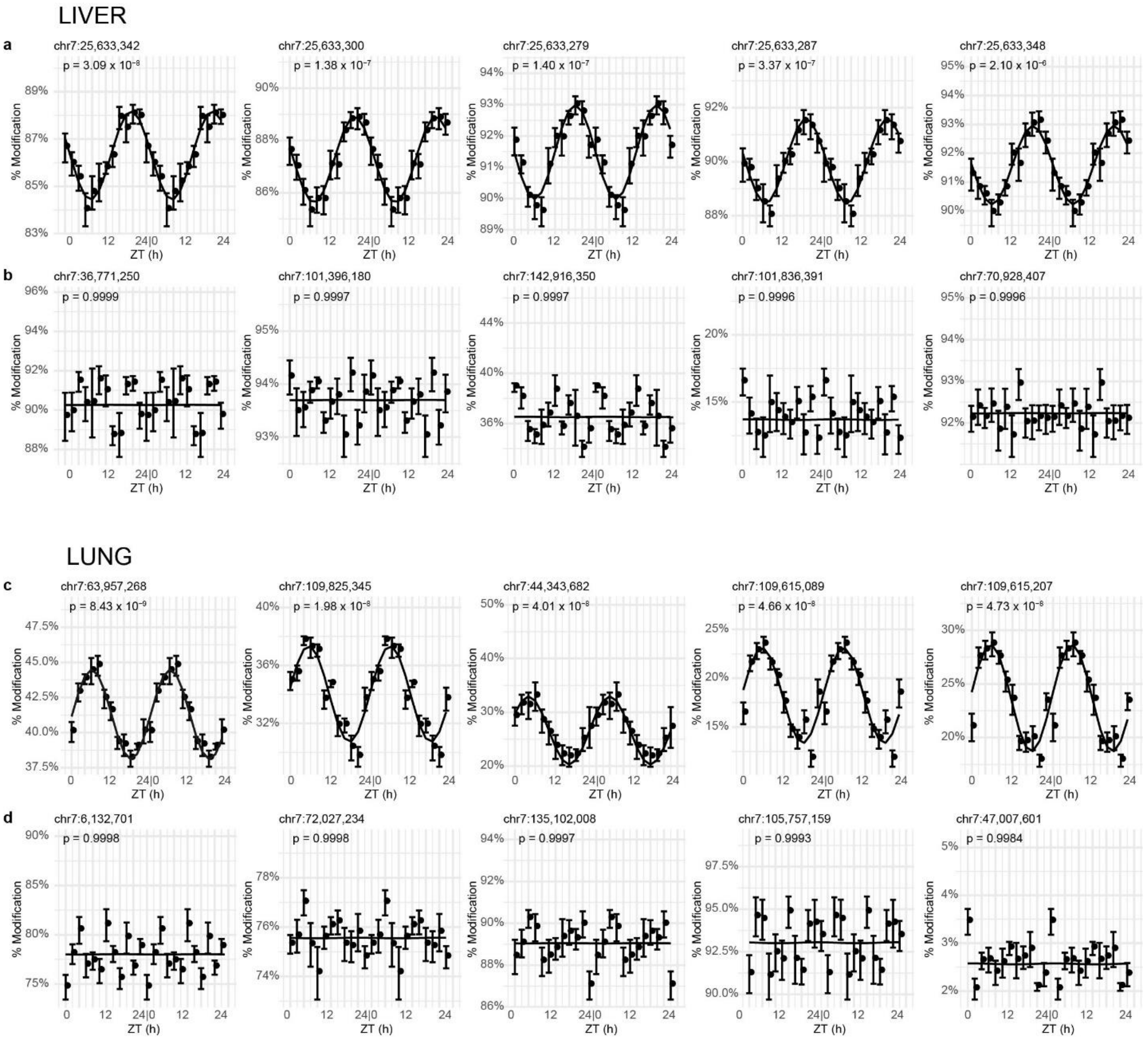

Supplementary Figure 2. Representative osc-modCs from mouse 9-mo liver and lung. The top 5 sites (ranked based on p-values) displaying oscillations of cytosine modification in (a) the liver and (c) the lung. Each point represents the mean of 3 biological replicates (where each biological replicate is represented by the median of 3 technical replicates) and error bars represent standard error of the mean (SEM). The coordinates for each CpG and the harmonic regression p-value are indicated at the top of each plot. The bottom 5 CpGs in (b) the liver and (d) the lung. ZT, Zeitgeber time.

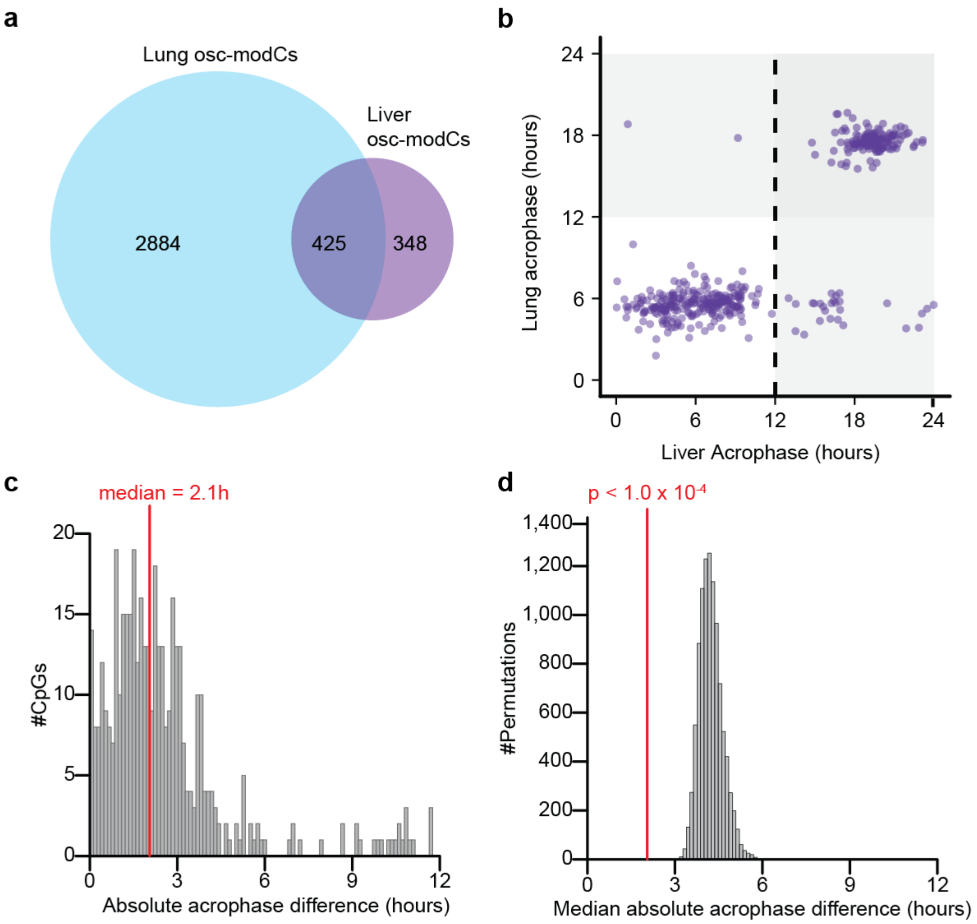

Supplementary Figure 3. Similarities between overlapping osc-modCs of the mouse 9-mo liver and lung. a) Venn diagram showing overlapping osc-modCs between the liver and the lung for epigenetically variable cytosines that are common between the two tissues. b) Scatter plot of acrophases of common osc-modCs in the liver and the lung. The shaded areas depict periods of lights off and the unshaded areas show periods of lights on. c) The absolute acrophase differences (minor arc length) between common liver and lung osc-modCs. The red line represents the median absolute acrophase difference. d) The distribution of expected median absolute acrophase differences obtained by permuting acrophases of common osc-modCs in the liver and lung. The red line represents the observed value depicted in (c). Osc-modC, oscillating modified cytosines.

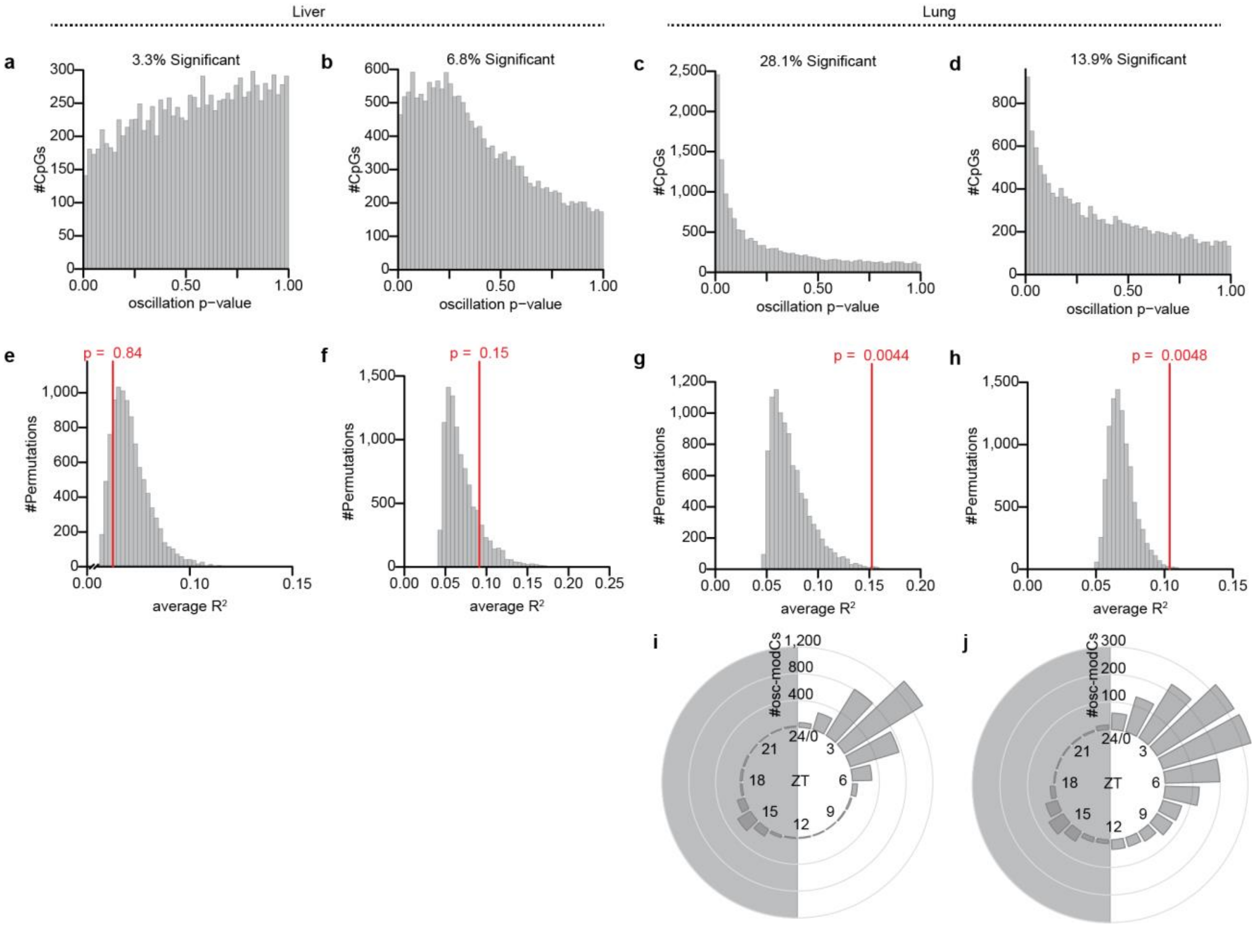

Supplementary Figure 4. Characterization of oscillating modified cytosines in the 15-mo and 25-mo mouse liver and lung. a-d) Histogram of harmonic regression p-values with the proportion of oscillating modified cytosines ($p < 0.05$) in the liver (a) 15-mo and (b) 25-mo, and the lung (c) 15-mo and (d) 25-mo. e-h) Average proportion of variance explained (R^2) by the harmonic regression fits across all investigated cytosines in each of 10,000 permutations of ZT labels. The red line depicts the observed average R^2 in the liver (e) 15-mo and (f) 25-mo, and the lung (g) 15-mo and (h) 25-mo. i-j) Acrophase rose plot showing modification peak times of osc-modCs in the lung (i) 15-mo and (j) 25-mo. Only epigenetically variable cytosines were investigated in (a-j). ZT, Zeitgeber time; osc-modCs, oscillating cytosine modifications; mo, month-old.

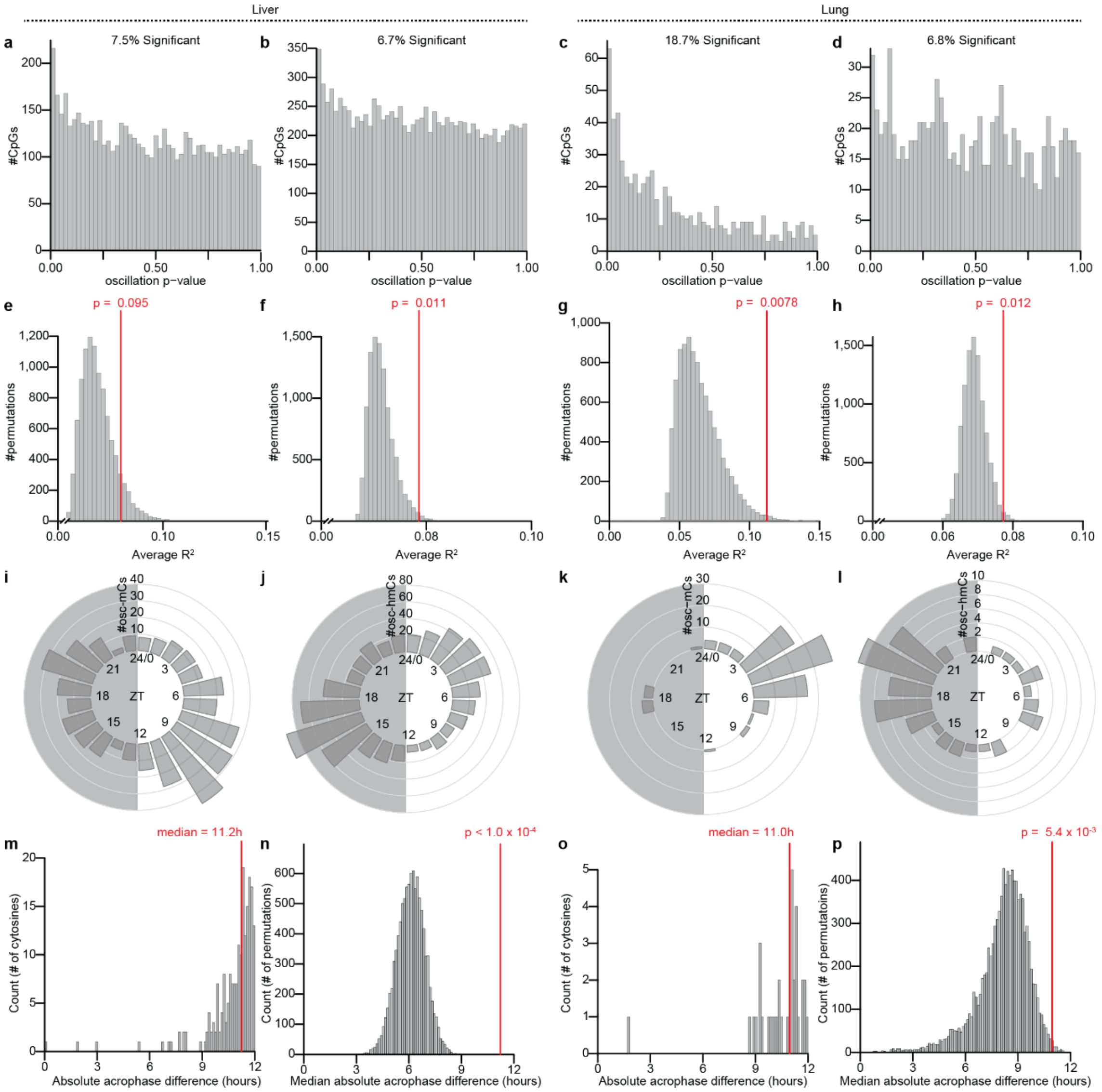

Supplementary Figure 5. Characterization of oscillating methylated and hydroxymethylated cytosines in the 9-mo mouse liver and lung. a-d) Histogram of harmonic regression p-values with the proportion of oscillating mCs and hmCs ($p < 0.05$) in (a-b) the liver and in (c-d) the lung. e-h) Average proportion of variance explained (R^2) by the harmonic regression fits across all interrogated cytosines in each of 10,000 permutations of ZT labels. The red line depicts the observed average R^2 of mC and hmC in (e-f) the liver and in (g-h) the lung. i-l) Acrophase rose plot showing peak modification times of mC and hmC oscillations in (i-j) the liver and in (k-l) the lung. m-p) The absolute acrophase differences (minor arc length) between commonly oscillating mC and hmC in (m) the liver and (o) the lung. The red line represents the median absolute acrophase difference. The distribution of the expected median absolute acrophase differences obtained by permuting acrophase pairs of common oscillating mCs and hmCs in (n) the liver and (p) the lung. The red line represents the observed value depicted previously in (m) and (o), respectively. Only epigenetically variable cytosines were investigated in (a-p). ZT, Zeitgeber time; Osc-mC, oscillating 5-methylcytosine; Osc-hmC, oscillating 5-hydroxymethylcytosine.

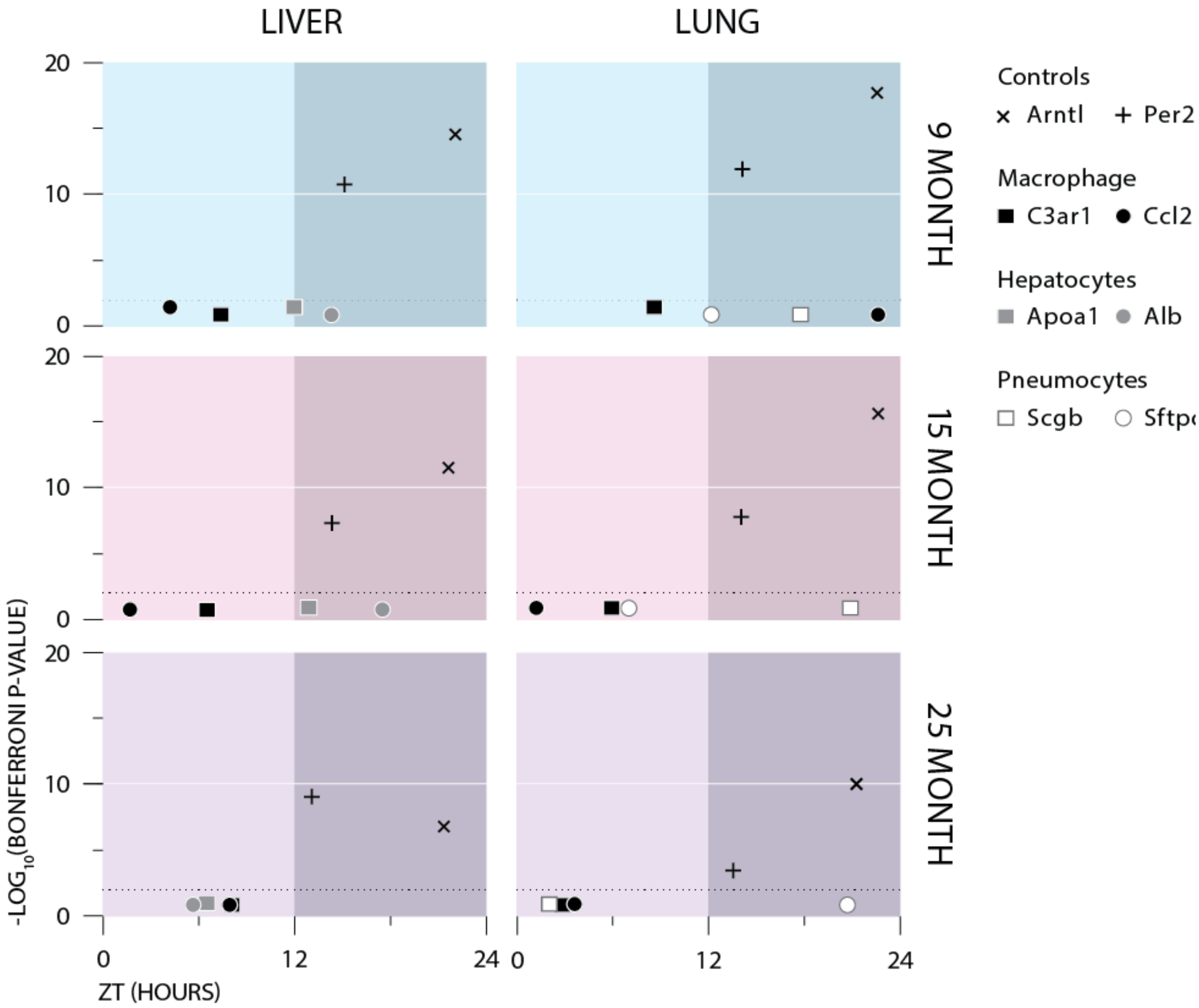

Supplementary Figure 6. RT-qPCR analysis of non-oscillating tissue-specific mRNA in mouse liver and lung tissues. Relative mRNA levels of the following six genes were measured as a function of time: I) albumin (*Alb*) and apolipoprotein a1 (*Apoa1*) representing hepatocytes; II) secretoglobin family 3a member 2 (*Scgb3a2*) and surfactant protein c (*Sftpc*) representing pneumocytes; and III) complement c3a receptor 1 (*C3ar1*); and c-c motif chemokine ligand 2 (*Ccl2*) representing macrophages. The figure displays Bonferroni-corrected p-values of the harmonic regression fits for each mRNA plotted across the acrophase time with the line marking the p = 0.05 significance threshold. ZT, Zeitgeber time.

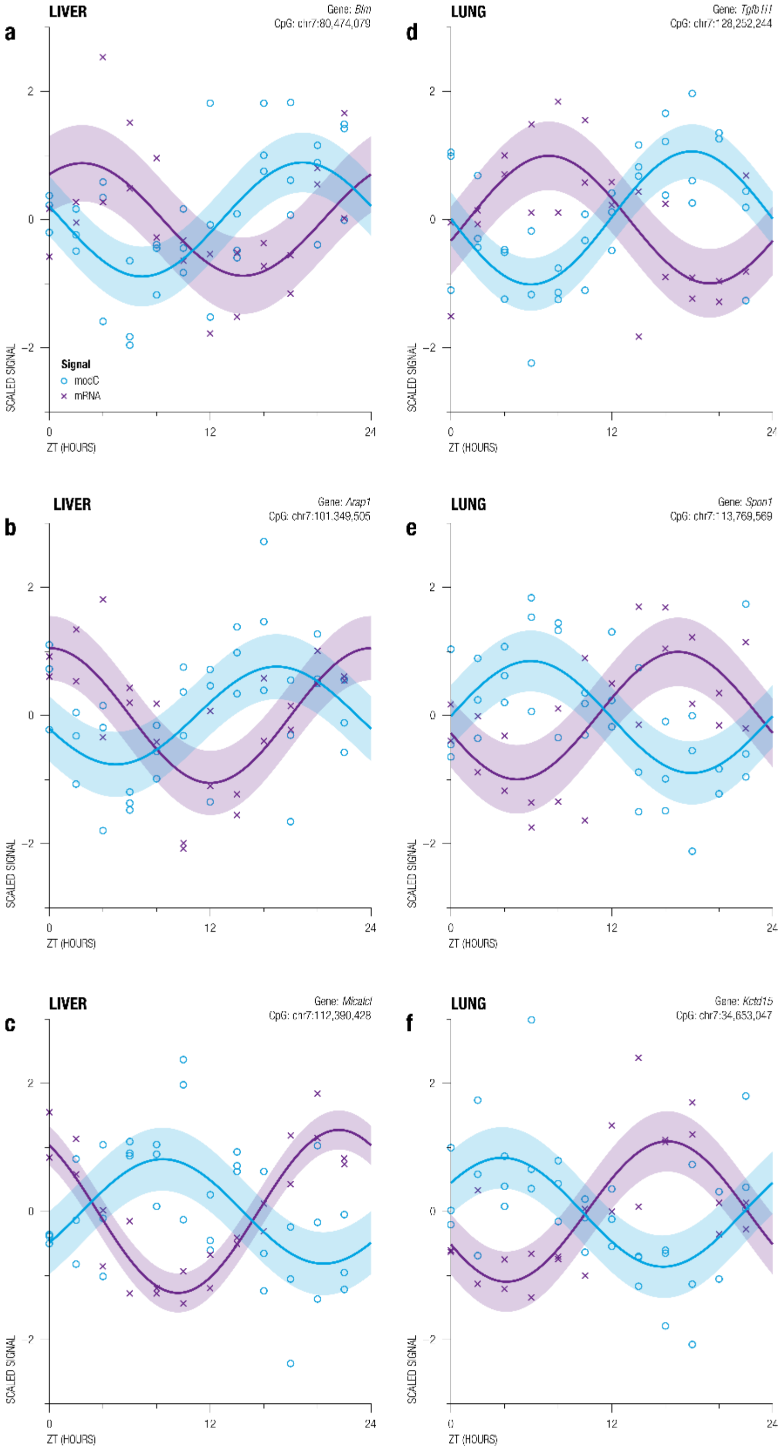

Supplementary Figure 7. Selected examples of the relationship between osc-modC and oscillating mRNA. Examples of osc-modC sites that overlap with oscillating mRNA in (a-c) the liver and (d-f) the lung. The sites were selected based on their harmonic regression p-values ($p < 0.005$) and high correlation between cytosine modification and mRNA expression to demonstrate the phase relationship between the two. The data was scaled to mean of 0 and standard deviation of 1. The shading around the regression line represents the 95% confidence band. Osc-modC, oscillating modified cytosines; ZT, Zeitgeber time.

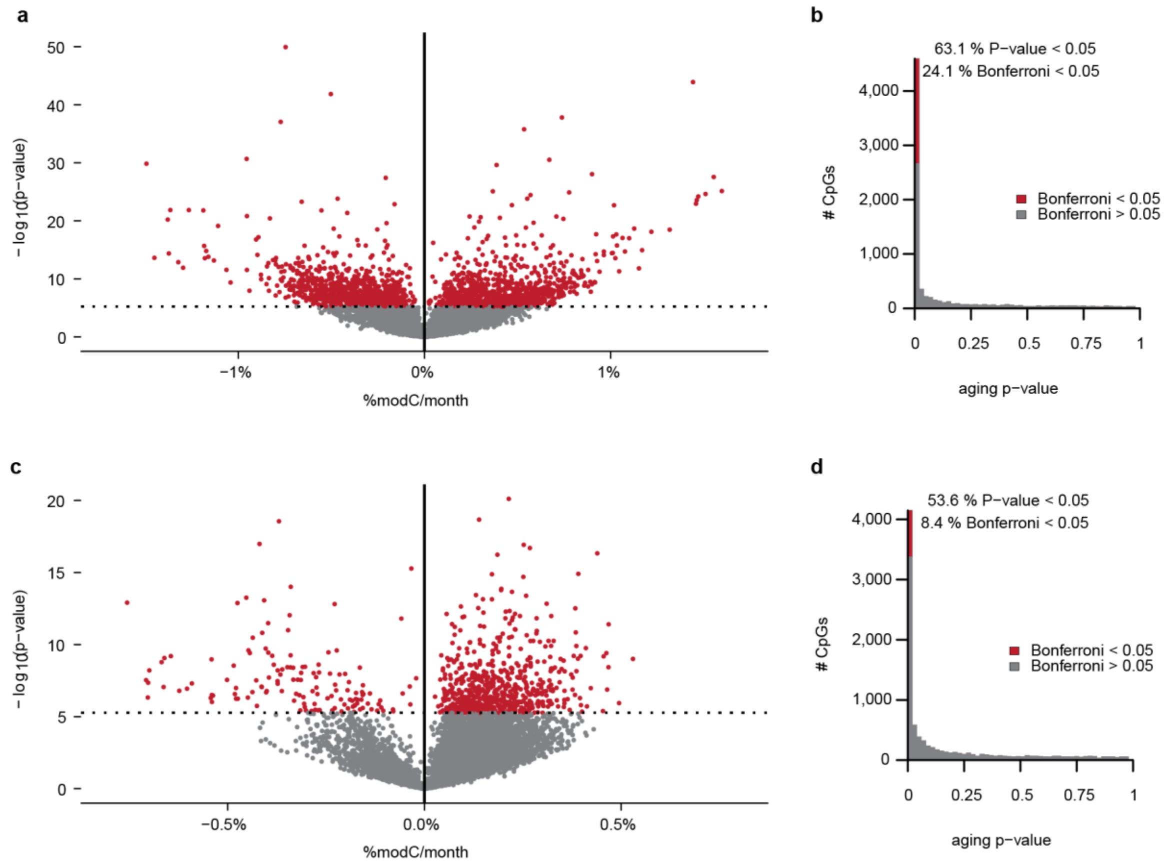

Supplementary Figure 8. Age- dependent cytosine modification changes in mouse tissues. a-d) Volcano plot illustrating the direction, magnitude, and statistical significance of age-dependent cytosine modification changes in (a) the liver and (c) the lung, and their corresponding p-value histogram of linear regression fits using age as a predictor across all mouse age cohorts in (b) the liver and (d) the lung. 24.1% (1933 out of 8011) and 8.4% (770 out of 9212) of the interrogated cytosines passed this significance threshold in the liver and lung, respectively. Bonferroni corrected significance threshold (p = 0.05) is indicated by the dotted line in the volcano plots. Only EVCs common across all age cohorts were investigated.

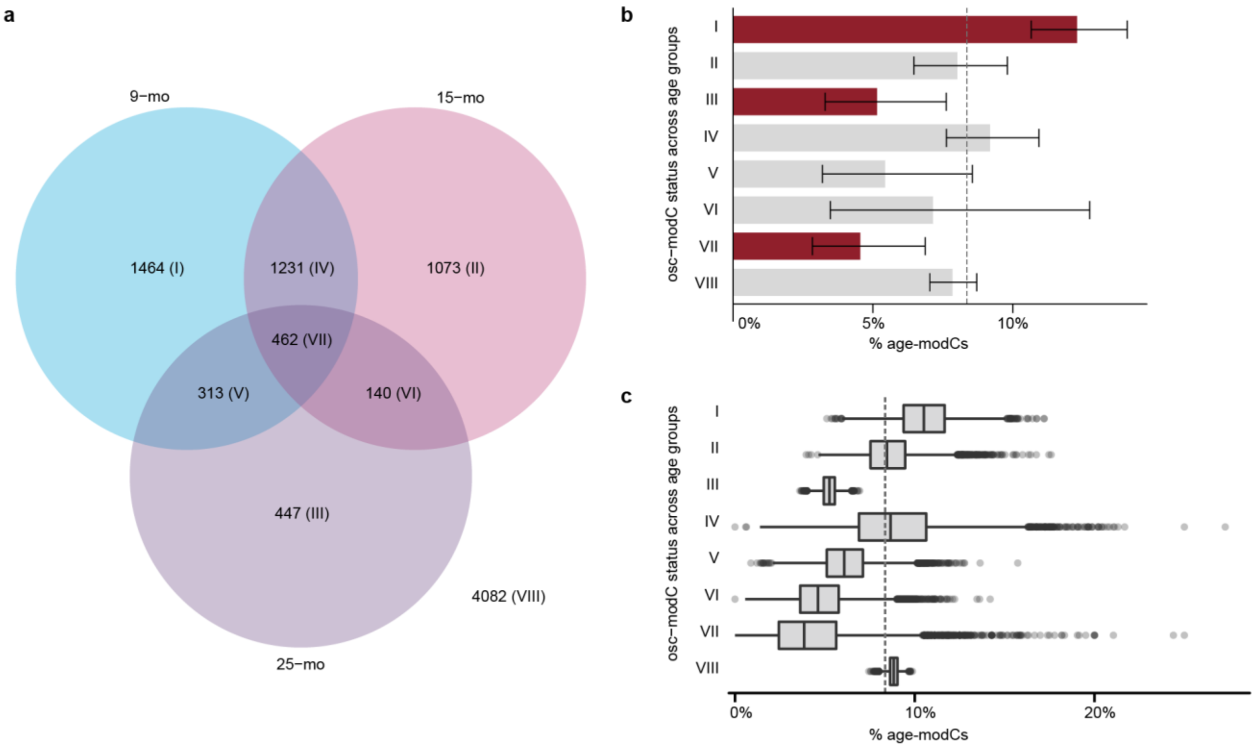

Supplementary Figure 9. osc-modC overlap with age-modC after adjusting for sample size or proportion of osc-modCs in the mouse lung. a) A Venn diagram of osc-modCs in the three age cohorts, where all age cohorts were matched to have the same sample size (n=28) and harmonic regression analysis was repeated for each. The various elements in the Venn diagram that represents groups of osc-modC with specific overlapping properties are indicated by roman numerals with the number of osc-modCs next to it. b) Bar plots showing the percentage of cytosines in each osc-modC category, illustrated in (a), that were age-modCs. The red bars indicate significant enrichment or de-enrichment of age-modCs. The error bars show 95% confidence intervals, and the dashed line represents the proportion of age-modCs relative to all epigenetically variable cytosines (i.e. chance level of age-modC proportions). c) Boxplots of 10,000 permutations showing the percentage of cytosines in each osc-modC category that were age-modCs, where proportion of osc-modC in all three age groups were matched by iteratively finding a sample size for each group that results in a similar number of osc-modCs. Osc-modC, oscillating modified cytosines; Age-modC, age-correlated cytosine modifications

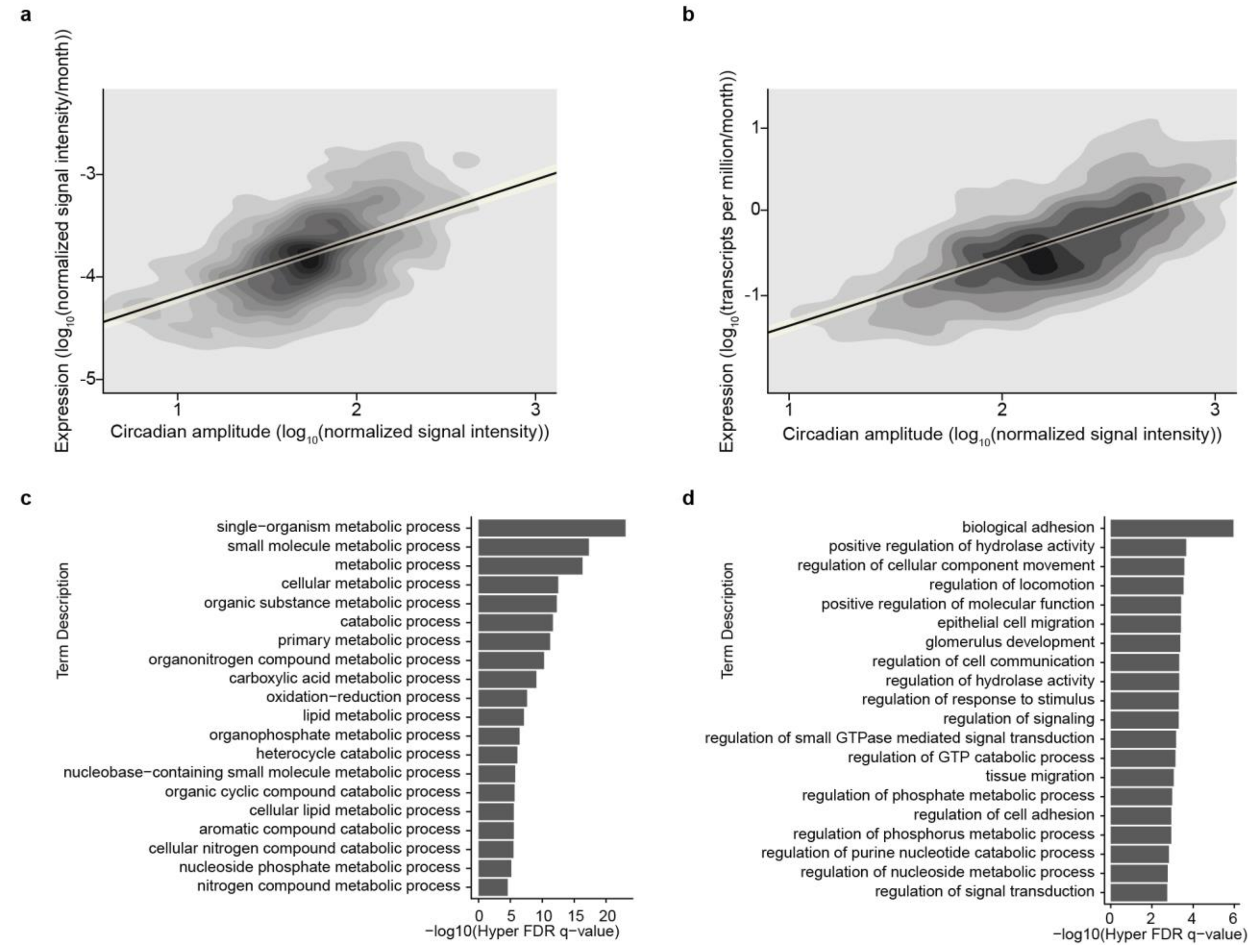

Supplementary Figure 10. Age dependent mRNA changes as a function of mRNA oscillation in the mouse liver and lung. a-b) Density contour plots of the magnitude of age-dependent mRNA changes and the amplitude of circadian oscillation from independent public datasets for matching genes in (a) the liver[1,2] (Pearson's r = 0.65, p = 4.4 × 10^{-170}) and (b) the lung[1,3] (Pearson's r = 0.56, p = 9.4 × 10^{-83}). Contours were rendered using 2D kernel density estimation, and the lines represent linear regression fits. Shading around the regression lines represents the 95% confidence band. c-d) Top 20 Gene Ontology terms (biological processes) significantly enriched in genes with with significant oscillation and aging in (c) the liver and (d) the lung.

Supplementary Table 1. Effects of matched sample size on the oscillation parameters for the mouse liver and lung tissues

Age	Tissue	Permuted p-value	% osc-modC	N
9-mo	Liver	0.057	7.46	28
15-mo	Liver	0.91	3.15	28
25-mo	Liver	0.19	6.60	28
9-mo	Lung	<0.0001	31.92	27
15-mo	Lung	0.0051	26.60	27
25-mo	Lung	0.0078	12.35	27

Supplementary Table 2. Oscillation parameters of tissue specific transcripts in mouse tissues

Acrophase	Adjusted p-value	Unadjusted p-value	Age	Tissue	Gene	Sample
14.556	1	0.133004	9-mo	Primary	*Alb*	Liver
12.097	0.427981	0.011888	9-mo	Primary	*Apoa1*	Liver
6.9907	1	0.162811	9-mo	Macrophage	*C3ar1*	Liver
3.4693	0.261081	0.007252	9-mo	Macrophage	*Ccl2*	Liver
8.6792	0.451567	0.012544	9-mo	Tcell	*Cxcr6*	Liver
12	1	1	9-mo	All	*Gapdh*	Liver
18.019	1	0.410607	15-mo	Primary	*Alb*	Liver
13.021	1	0.104658	15-mo	Primary	*Apoa1*	Liver
5.8787	1	0.310868	15-mo	Macrophage	*C3ar1*	Liver
0.6382	0.937219	0.026034	15-mo	Macrophage	*Ccl2*	Liver
10.636	1	0.137479	15-mo	Tcell	*Cxcr6*	Liver
12	1	1	15-mo	All	*Gapdh*	Liver
5.0028	1	0.409222	25-mo	Primary	*Alb*	Liver
5.9565	1	0.294842	25-mo	Primary	*Apoa1*	Liver
7.6835	1	0.100556	25-mo	Macrophage	*C3ar1*	Liver
7.5184	1	0.396406	25-mo	Macrophage	*Ccl2*	Liver
6.651	1	0.032476	25-mo	Tcell	*Cxcr6*	Liver
12	1	1	25-mo	All	*Gapdh*	Liver
23.1	3.2E-14	8.89E-16	9-mo	Control	*Arntl*	Liver
15.467	1.81E-10	5.02E-12	9-mo	Control	*Per2*	Liver
22.555	1.99E-11	5.53E-13	15-mo	Control	*Arntl*	Liver
14.578	3.31E-07	9.18E-09	15-mo	Control	*Per2*	Liver
22.295	1.36E-06	3.78E-08	25-mo	Control	*Arntl*	Liver
13.196	8.26E-09	2.29E-10	25-mo	Control	*Per2*	Liver
18.301	1	0.087336	9-mo	Primary	*Scgb*	Lung
12.202	1	0.255199	9-mo	Primary	*Sftpc*	Lung

8.318	0.271788	0.00755	9-mo	Macrophage	*C3ar1*	Lung
23.713	1	0.190666	9-mo	Macrophage	*Ccl2*	Lung
6.3581	1	0.059626	9-mo	Tcell	*Cxcr6*	Lung
12	1	1	9-mo	All	*Gapdh*	Lung
21.688	1	0.490392	15-mo	Primary	*Scgb*	Lung
6.5755	1	0.566351	15-mo	Primary	*Sftpc*	Lung
5.295	1	0.282577	15-mo	Macrophage	*C3ar1*	Lung
0.1587	1	0.03835	15-mo	Macrophage	*Ccl2*	Lung
3.8897	0.070944	0.001971	15-mo	Tcell	*Cxcr6*	Lung
12	1	1	15-mo	All	*Gapdh*	Lung
1.114	1	0.314925	25-mo	Primary	*Scgb*	Lung
21.533	1	0.147431	25-mo	Primary	*Sftpc*	Lung
1.8807	1	0.498463	25-mo	Macrophage	*C3ar1*	Lung
2.8328	1	0.244947	25-mo	Macrophage	*Ccl2*	Lung
0.6687	1	0.030816	25-mo	Tcell	*Cxcr6*	Lung
12	1	1	25-mo	All	*Gapdh*	Lung
23.575	2.43E-17	6.75E-19	9-mo	Control	*Arntl*	Lung
14.343	1.21E-11	3.37E-13	9-mo	Control	*Per2*	Lung
23.659	2.48E-15	6.88E-17	15-mo	Control	*Arntl*	Lung
14.211	1.39E-07	3.86E-09	15-mo	Control	*Per2*	Lung
22.152	9.04E-10	2.51E-11	25-mo	Control	*Arntl*	Lung
13.712	0.00249	6.92E-05	25-mo	Control	*Per2*	Lung

Supplementary Table 3. Primer list for qPCR

Gene	Forward Primer	Reverse Primer
Gapdh	TGCACCACCAACTGCTTAGC	GCATGGACTGTGGTCATGAG
Per2	TCACCCCAGCCCTGATGAT	ACTGCTCACTACTGCAGCC
Arntl	AATGCAAGGGAGGCCCACA	CCATCCTTAGCACGGTGAGT
Apoa1	AACAGCTGAACCTGAATCTCCT	CCCAATCTGTTTCTTTCTCCAG
Alb	TGGATGACTTTGCACAGTTCCT	AAGGTTTGGACCCTCAGTCG
Ccl2	GCTGTAGTTTTTGTCACCAAGC	GCTGAAGACCTTAGGGCAGA
Sftpc	GTCCTTGAGATGAGCATCGG	CAGGAGCCGCTGGTAGTC
Scgb	CCATCATTTGAGGCTCTTTCAC	CTAGACTCCTTAAACTCTGGGA
C3ar1	TGGTTTCTCCAGTGCCCAGT	GCCTTTTCTTCCTGTCTACAAAG

Supplementary References

1 Zhang, R., Lahens, N. F., Ballance, H. I., Hughes, M. E. & Hogenesch, J. B. A circadian gene expression atlas in mammals: implications for biology and medicine. *Proc Natl Acad Sci U S A* **111**, 16219-16224, doi:10.1073/pnas.1408886111 (2014).

2 Bochkis, I. M., Przybylski, D., Chen, J. & Regev, A. Changes in nucleosome occupancy associated with metabolic alterations in aged mammalian liver. *Cell Rep* **9**, 996-1006, doi:10.1016/j.celrep.2014.09.048 (2014).

3 Misra, V. *et al.* Global expression profiles from C57BL/6J and DBA/2J mouse lungs to determine aging-related genes. *Physiol Genomics* **31**, 429-440, doi:10.1152/physiolgenomics.00060.2007 (2007).
