# Supplementary material for: Cytosine modifications exhibit circadian oscillations that are involved in epigenetic diversity and aging
Source: Nat Commun. 2018 Feb 13;9:644. doi: 10.1038/s41467-018-03073-7 (PMC5811577; doi:10.1038/s41467-018-03073-7)
Supplement: Supplementary file 3 — Description of Additional Supplementary Files [file 41467_2018_3073_MOESM3_ESM.pdf]

Description of Additional Supplementary Files

File Name: Supplementary Data 1
Description: Genomic coordinates and sequences targeted by padlock probes.

File Name: Supplementary Data 2
Description: Genomic coordinates and oscillation parameters of the bisulfite padlock sequencing for 9-mo liver.

File Name: Supplementary Data 3
Description: Genomic coordinates and oscillation parameters of the bisulfite padlock sequencing for 9-mo lung.

File Name: Supplementary Data 4
Description: Genomic coordinates and oscillation parameters of the bisulfite padlock sequencing for 15-mo lung.

File Name: Supplementary Data 5
Description: Genomic coordinates and oscillation parameters of the bisulfite padlock sequencing for 25-mo lung.

File Name: Supplementary Data 6
Description: Genomic coordinates and oscillation parameters of the bisulfite padlock sequencing for 15-mo liver.

File Name: Supplementary Data 7
Description: Genomic coordinates and oscillation parameters of the bisulfite padlock sequencing for 25-mo liver.

File Name: Supplementary Data 8
Description: Genomic coordinates and oscillation parameters of the oxidative bisulfite padlock sequencing for 9-mo liver.

File Name: Supplementary Data 9
Description: Genomic coordinates and oscillation parameters of 5- hydroxymethylcytosines for 9-mo liver.

File Name: Supplementary Data 10
Description: Genomic coordinates and oscillation parameters of the oxidative bisulfite padlock sequencing for 9-mo lung.

File Name: Supplementary Data 11
Description: Genomic coordinates and oscillation parameters of 5- hydroxymethylcytosines for 9-mo lung.

File Name: Supplementary Data 12
Description: Oscillation parameters of public mouse liver mRNA.

File Name: Supplementary Data 13
Description: Oscillation parameters of public mouse lung mRNA.

File Name: Supplementary Data 14
Description: MEME output for the oscillating cytosines in the liver tissue from 9-mo mice.

File Name: Supplementary Data 15
Description: MEME output for the oscillating cytosines in the lung tissue from 9-mo mice.

File Name: Supplementary Data 16
Description: Aging parameters for the mouse liver dataset from bisulfite padlock sequencing.

File Name: Supplementary Data 17
Description: Aging parameters for the mouse lung dataset from bisulfite padlock sequencing.

File Name: Supplementary Data 18
Description: Aging parameters of public mouse liver mRNA.

File Name: Supplementary Data 19
Description: Aging parameters of public mouse lung mRNA.

File Name: Supplementary Data 20
Description: Gene ontology enrichment analysis of the oscillating-aging associated transcripts from the liver transcriptome datasets.

File Name: Supplementary Data 21
Description: Gene ontology enrichment analysis of the oscillating-aging associated transcripts from the lung transcriptome datasets.

File Name: Supplementary Data 22
Description: Primer list for sequencing library preparation.
